# Growth and Heavy Metal Accumulation of *Koelreuteria Paniculata* Seedlings and Their Potential for Restoring Manganese Mine Wastelands in Hunan, China

**DOI:** 10.3390/ijerph120201726

**Published:** 2015-02-03

**Authors:** Zhihong Huang, Wenhua Xiang, Yu’e Ma, Pifeng Lei, Dalun Tian, Xiangwen Deng, Wende Yan, Xi Fang

**Affiliations:** 1 National Engineering Laboratory for Applied Technology of Forestry & Ecology in South China, Central South University of Forestry and Technology, Changsha 410004, Hunan, China; E-Mails: huanghugh2013@yahoo.com (Z.H.); pifeng.lei@gmail.com (P.L.); csufttdl@126.com (D.T.); dxwfree@126.com (X.D.); csfuywd@hotmail.com (W.Y.); fangxizhang@sina.com (X.F.); 2 Faculty of Life Science and Technology, Central South University of Forestry and Technology, Changsha 410004, Hunan, China; E-Mail: yuema2013@hotmail.com

**Keywords:** *Koelreuteria paniculata*, lateral fine root development, seedling biomass, phytoremediation, manganese mine wastes

## Abstract

The planting of trees on mine wastelands is an effective, long-term technique for phytoremediation of heavy metal-contaminated wastes. In this study, a pot experiment with seedlings of *Koelreuteria paniculata* under six treatments of local mine wastes was designed to determine the major constraints on tree establishment and to evaluate the feasibility of planting *K. paniculata* on manganese mine wastelands. Results showed that *K*. *paniculata* grew well in mine tailings, and also under a regime of equal amounts of mine tailings and soil provided in adjacent halves of pots. In contrast, mine sludge did not favor survival and growth because its clay texture limited fine root development. The bio-concentration factor and the translocation factor were mostly less than 1, indicating a low phytoextraction potential for *K*. *paniculata*. *K*. *paniculata* is suited to restore manganese mine sludge by mixing the mine sludge with local mine tailings or soil.

## 1. Introduction

Recent studies report that exposure to manganese (Mn) results in neurotoxicity and/or Parkinson’s disease (PD) in welders [1] and nurses [2]. This phenomenon has aroused widespread concern on a global scope [3,4,5]. Manganese-induced clinical neurotoxicity is associated with a motor dysfunction syndrome commonly referred to as manganism [6], which is related with significantly higher reactive oxygen species (ROS) generation [7]. It is therefore great concern about the environmental risk posed by manganese waste mines because most of the tailings have been left without any management and have become the main source of heavy metal contamination of agricultural soils and crops in the mining areas [8]. Soil heavy metal pollution poses high carcinogenic and non-carcinogenic risks to the public, especially to children and those living in the vicinity of heavily polluted mining areas [9,10]. Multiple pathways of health risk due to heavy metal exposure in China were reviewed by Zhuang *et al.* [11], with risks coming from the intake of home-grown rice and vegetables. The use of polluted groundwater and pond water also poses potential risk to human health [12], for instance, the high manganese concentration (1.21 ppm; U.S. Environmental Protection Agency reference, <0.05 ppm) in water used by local residents had caused a markedly below-average performance in tests of memory [13] due to human nervous system damage cuased by excessive intake of manganese [14]. Chai *et al.* [15] found that arsenic and manganese were the largest contributors to human health risks for the local people drinking groundwater in the Xiangjiang watershed. The neurotoxicologic effects of water manganese in children [16] due to their significantly higher Mn concentrations in blood (9.5 μg/L) and hair (12.6 μg/L) was observed [17]. Furthermore, water Mn concentrations of 0.66 μg/L [18] (*i.e*., higher than the WHO guideline of <0.4 μg/L) may lead to higher infant mortality [19].

In China, mining activities alone have resulted in the creation of about 3.2 million ha of wastelands; and this figure keeps on increasing at a rate of 46,700 ha per year [20], due to the rapid expansion of the mining industry in order to meet the demands of rapid economic growth. Unlike organic compounds heavy metals cannot be degraded, so the clean-up of heavy metals usually requires removal or immobilization [21]. Recently, a number of instances of food contamination and of health problems amongst local residents caused by heavy metals in the environment have been reported in China [22]. Although much effort and attention has been paid to the problem by governments at every level, only a low rate of restoration of metalliferous mine wastelands has so far been achieved, making it crucial to develop efficient techniques for the restoration of wastelands associated with heavy metals [20].

Compared to treatments that involve engineering or physicochemical techniques, phytoremediation is a low-cost and environmentally benign “green” technology that is also easily translated to large-scale applications [23]. Plants not only have the capacity to remove pollutants from mine wastelands or to render them harmless [24], but they also maintain the biological activity and physical structure of soils [25]. The two main categories of processes for the phytoremediation of soils contaminated with heavy metals are phytoextraction and phytostabilization [26]. Phytoextraction usually uses plants to take up heavy metals from the soil and to translocate them from the roots to the above-ground parts [27]. Obviously, in order to achieve effective soil decontamination, the basic requirement for phytoextraction is that plants should exhibit high productivity, and that they should be able to accumulate and tolerate high concentrations of heavy metals [28]. For this reason, the selection of hyperaccumulators that are effective for specific heavy metals has become a priority [29]; however, most of those that have been selected are herbs that have low biomass and shallow root systems, so that their ability to achieve substantial heavy metal accumulation and to deliver remediation effects at the deeper levels within soils are quite limited. By contrast, phytostabilization uses plants to retain heavy metals within the soil or within roots, or to reduce heavy-metal mobility and bioavailability [26]. Planting trees on mine wastes is promoted as a sustainable and ecologically sound solution for the phytostabilization of heavy-metal-contaminated soils [26,30]; and because natural restoration processes are rather slow [31], it is also used to accelerate the recovery of vegetation [32]. The benefits and advantages that result from tree planting include: soil stabilization and improvement [30], the accumulation or partial removal of heavy metals [26], carbon sequestration [33], renewable bioenergy production without detriment to the food chain or to human health [21], the provision of wildlife habitats and the conservation of biodiversity [34] and, perhaps most conspicuously, landscape amelioration and beautification.

The establishment of trees on mine wasteland is usually constrained by a physical soil structure that is unfavorable for water retention, aeration, and root penetration [35], also accompanied by poor nutrient status or by heavy metal toxicity [32]. Several studies have shown that many tree species can generally survive in metal-contaminated soils, though usually at a much reduced rate of growth [26]. Encouragement of the proliferation of fine roots in uncontaminated zones of the soil is an important strategy to avoid heavy metal toxicity and other stresses [36]. To improve the physicochemical and biological properties of mine wastelands and to increase tree survival and growth, applications of fertilizer and the incorporation of sand [32,37], along with physical manipulation of the mine wastes [30], are effective courses of action. The use of fertilizer and sand to improve a large area of mine wasteland is expensive, however, both in terms of the costs of the materials themselves and in transportation costs. It remains unclear whether, as an alternative or complementary approach, the manipulation of local mine wastes with contrasting physical and chemical properties could be used to create heterogeneity in wastelands and thereby facilitate tree survival and growth.

The Xiangtan manganese (Mn) mine is one of the most important metal mines in Hunan Province. It has been in operation for more than 80 years and left large areas of abandoned mine wastelands. These wastelands still exist and are grouped into mine tailings and mine sludge. Mine tailings are characterized by low nutrient status and a silt-like texture [29], while mine sludge represents a nutrient-rich substrate that is favorable for revegetation, which major disadvantages in terms of plant establishment are its degree of compaction and its anoxic nature [38].

When grown on mine tailings, the herb species *Phytolacca acinosa* has been found to be a Mn hyperaccumulator [29]. In addition, on account of its fast growth rate and high adaptive capacity, the tree species *Koelreuteria paniculata* has demonstrated a potential for phytoremediation [39] and has been successfully established on mine tailings, with a *ca.* 96% survival rate. Nevertheless, the mechanism of the phytoremediation of mine tailings brought about by *K*. *paniculata* trees is uncertain; and furthermore, it is unclear whether local waste manipulations, for example, through mixing mine tailings with mine sludge, might facilitate the survival and growth of *K*. *paniculata* in mine sludge. The objectives of the study reported here were therefore: (1) to investigate the limitations of mine sludge, compared to mine tailings, in the growth of *K*. *paniculata* seedlings; (2) to evaluate whether local waste manipulations of mine tailings and mine sludge could improve the survival rate and growth of *K*. *paniculata* seedlings; and (3) to determine the heavy-metal phytoremediation mechanism of *K*. *paniculata* in Mn mine wastelands.

## 2. Materials and Methods

### 2.1. Experimental Materials

Samples of mine tailings and mine sludge for pot-grown plants were collected from the Xiangtan Mn mine (27°53′–28°03′ N, 112°45′–112°55′ E), located in the northern part of the city of Xiangtan, Hunan Province, China. This region has a subtropical monsoon climate, with mean annual rainfall of 1431 mm and mean annual air temperature of 17.4 °C. Mine tailings are the abandoned wastes produced as a result of mineral processing and their area amounts to 134 ha. Mine sludge is the final mixture discharged into the tailing pond following the preliminary treatment of the manganese ore powder and its area is about 100 ha. Samples of about 50 kg each of mine tailings and mine sludge were randomly collected and transported to the laboratory for chemical analysis and for use in the experimental growth media.

Soils for use in a control experiment were sampled from a *Cinnamomum camphora* plantation in the campus of the Central South University of Forestry and Technology (CSUFT) (28°08′ N, 113°00′ E), Changsha City, Hunan Province, China. This site is hilly and also experiences a subtropical monsoon climate. The soil is red in color and it is derived from a plinthitic horizon soil parent material of the Quaternary period, and classified within Alliti-Udic ferrosols, which corresponds to acrisol in the World Reference Base for Soil Resources.

*K*. *paniculata* seedlings were sourced from the Zhuzhou nursery garden of CSUFT (27°54′ N, 113°09′ E). All seedlings were 1-year-old and of similar size: *ca.* 75 cm high, with a basal diameter of *ca.* 0.5 cm. The seedlings were selected immediately prior to being transplanted into experimental pots.

### 2.2. Experimental Design and Growth Media

**Figure 1:**
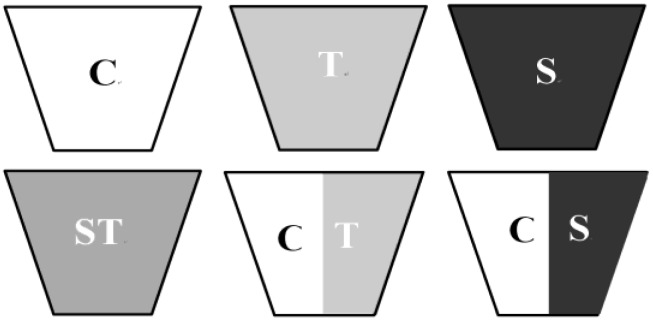
The experimental setup included six treatments, which were filled with different soil substrates in the pots. Each pot was labeled with the treatment code.

We designed six experimental treatments: (1) uncontaminated soil, as a control (C); (2) mine tailings (T); (3) mine sludge (S); (4) a 1:1 (v/v) mixture of mine tailings and mine sludge (ST); (5) a half-volume of mine tailings and a half-volume of soil (CT); and finally; (6) a half-volume of mine sludge and a half-volume of soil (CS). For treatments (5) and (6), the half-volumes of the two components were not mixed together, but were placed separately in each half of the plant pot. Therefore, for treatments (5) and (6), the CT-C and CS-C represented for the half-volume of soil of the treatments CT and CS, and the CT-T and CS-S were for the half of the pot filled with tailings and for the half filled with mine sludge, respectively. Treatments (5) and (6) were designed to test whether heterogeneity substrate could improve seedling growth and thus facilitate restoration in mine wastes. The details of the pots filled with different substrates were presented in Figure 1.

For each treatment, 12 PVC pots (32 cm high, top diameter 36 cm, bottom diameter 28 cm), with holes for drainage at the bottom, were prepared, which were sufficient for three harvesting times, each with four replicates.

**Figure 2:**
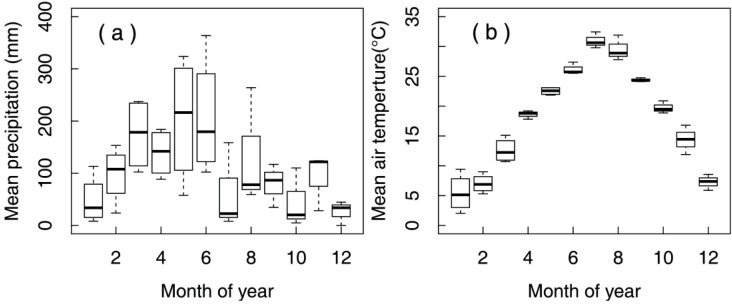
The box-plot for average monthly rainfall (**a**) and monthly air temperature (**b**).

After measurements had been made of the basal diameter, height and fresh weight of each *K*. *paniculata* seedling, and of the number of roots and their length, a seedling was placed centrally in each pot and affixed using two pieces of upright cardboard so as to ensure that the roots were straightened and spread out. Each pot was filled to within 2 cm of the rim, and when filling and planting were completed, the pieces of cardboard were carefully removed. After watering, the pots were then moved into a greenhouse shaded with warp-knitted netting. The growth of the seedlings was routinely monitored. The pots were watered at 2–3 d intervals to maintain soil moisture, and before each harvest measurement. The local climate conditions around the greenhouse were similar as the subtropical moonsoon climate as described above. Average monthly rainfall and air temperature were presented in Figure 2, based on the data recorded in microclimate observation station near the greenhouse during perdiod from 2010 to 2013.

### 2.3. Seedling Growth Measurements and Chemical Analysis

The pot experiments started in late March 2012. Three sets of measurements were taken, on separate groups of seedlings, in July, September and December. The measurements included determinations of survival rate, seedling height, basal diameter, fine root length and number, and shoot and root biomass. After taking measurements of height, basal diameter and leaf number, the seedlings were marked and then cut off at the base near the soil surface. The aerial parts were separated into woody shoots and leaves, respectively. The roots of the (marked) seedlings were washed carefully with tap water on 1 mm × 1 mm mesh and the root systems were collected. For each seedling, the number of roots and their length was determined. The samples of woody shoots, leaves and roots were washed thoroughly with running tap water, followed by three rinses with deionized water. They were then oven-dried at 80 °C and their dry mass was determined. After being ground into fine powder using a pestle and mortar, the samples were digested with a mixture of concentrated HNO_3_ and concentrated HClO_4_ (4:1, v/v), prior to chemical analysis.

Samples (~500 g) of soil, mine tailings, mine sludge, and the mine waste (tailings/sludge) mixture were taken before and after the experiment. pH values were measured potentiometrically with a pH meter (PXS-270, Shanghai, China), using a 1:2.5 (v/v) sample:H_2_O (distilled) suspension. Total nitrogen (N) concentrations were determined by a semimicro-Kjeldahl method. Total phosphorus (P) concentrations were measured using a sodium hydroxide-molybdenum-antimony colorimetric method [40]. Soil and mine waste samples were digested with HCl-HNO_3_-HF-HClO_4_. For the determination of the concentrations of metals in plant, soil and mine waste samples, the digested solutions were analyzed using a flame atomic absorption spectrophotometer (HP3510, Shanghai, China).

### 2.4. Data Analysis

To investigate the phytoremediation mechanism for heavy metals, the bio-concentration factor (BCF) was expressed as the ratio of the metal concentration in the above-ground part of the seedling to the total metal concentration in soil or mine wastes [38]. The translocation factor (TF) for metals within a given seedling was calculated as the metal concentration in the shoot divided by that in the root [26]. One-way ANOVA was used to test differences among treatments and Tukey-Kramer honest significant difference (HSD) tests were used for pair-wise comparisons of all treatments. Statistical significance was tested at the level of 0.05 using the JMP software package (SAS Institute Inc., Cary, NC, USA).

## 3. Results

### 3.1. Chemical Characteristics of Mine Wastes and Soil

The control soils used in this study shared some characteristics—acidity, clay content and low concentrations of nutrients—with the typical red soils of southern China. Compared to the control soils, the mine tailings were more alkaline (pH 9.07) and showed higher bulk density but lower nutrient (N, P and K) concentrations, whereas the pH of the mine sludge was close to neutral (pH 6.56) and it exhibited both higher bulk density and higher nutrient concentrations shown in Table 1.

### 3.2. Survival Rate and Fine Root Development

Survival rates for the *K*. *paniculata* seedlings were recorded after 7 to 15 days when the seedlings had been transplanted into their pots. All the seedlings (100%) survived under the C, T, and CT treatments. In contrast, the survival rate declined to 66.7% under the CS treatment and to 50% under the ST treatment; and no seedlings at all survived under the S treatment.

**Table 1:** Physical and chemical characteristics (mean value ± standard error) of soil (C), mine tailings (T), mine sludge (S), and the “a 1:1 mixture of mine tailings and mine sludge” (ST).

Samples	pH	Bulk Density (g∙cm^−3^)	Total N (g∙kg^−1^)	Total P (g∙kg^−1^)	Total K (g∙kg^−1^)	Fe (mg∙kg^−1^)	Zn (mg∙kg^−1^)	Mn (mg∙kg^−1^)
C	4.29	1.37	1.200 ± 0.082 ^a^	0.606 ± 0.046 ^b^	0.029 ± 0.001 ^a^	11,803.1 ± 69.1 ^b^	51.7 ± 9.6 ^c^	97.6 ± 16.3 ^c^
T	9.07	1.44	0.575 ± 0.150 ^b^	0.359 ± 0.344 ^b^	0.001 ± 0.001 ^b^	2396.7 ± 17.3 ^c^	61.0 ± 42.9 ^c^	13,178.3 ± 77.5 ^b^
S	6.56	1.64	1.275 ± 0.050 ^a^	4.083 ± 0.281 ^a^	0.018 ± 0.003 ^b^	17,185.7 ± 334.4 ^a^	170.0 ± 12.5 ^a^	17,391.4 ± 64.8 ^a^
ST	8.37	1.54	0.900 ± 0.000 ^b^	3.462 ± 0.062 ^a^	0.015 ± 0.001 ^b^	13,292.0 ± 5.4 ^b^	135.28.4 ^b^	16,484.0 ± 124.5 ^a^
Background value ^(1)^	−	−	−	−	−	34,000	94.9	320
Grade II ^(2)^	−	−	−	−	−	−	200	−

Note: ^(1)^ soil trace element background for Hunan Province from China National Environmental Monitoring Center, 1990; ^(2)^ values from the metal standards of China Soil Environmental Quality (GB15618-1995, Grade II). The lowercase letters (a, b, c) indicate significant differences among soil substrates of the treatments (*p* < 0.05).

**Figure 3:**
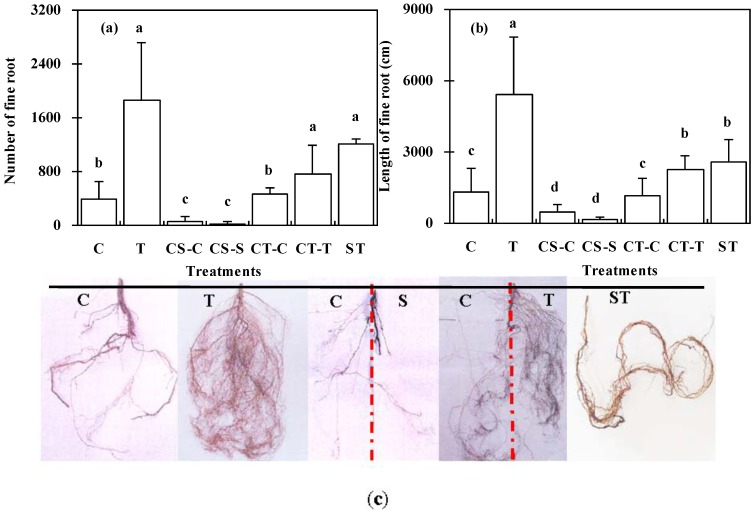
Total fine root number per plant (**a**), and root length (**b**), of *K*. *paniculata* seedlings grown under various treatments. Mean values with error bars are shown; Different letters indicate significant differences among treatments (*p* < 0.05); Representative images of fine roots development of seedlings under the different treatments are presented in panel (**c**). A broken red line was used to separate the different treatments of CS (-C/-S) and CT (-C/-T).

At the end of the pot experiments in December, both the number of fine roots and the total length of fine roots differed significantly among the various treatments (*p* < 0.05) shown in Figure 3. The highest values for both root number and root length were found under the T treatment, followed by the CT treatment and the ST treatment. The lowest values were obtained with the seedlings under the CS treatment.

### 3.3. Biomass Allocation of K. Paniculata Seedlings

**Figure 4:**
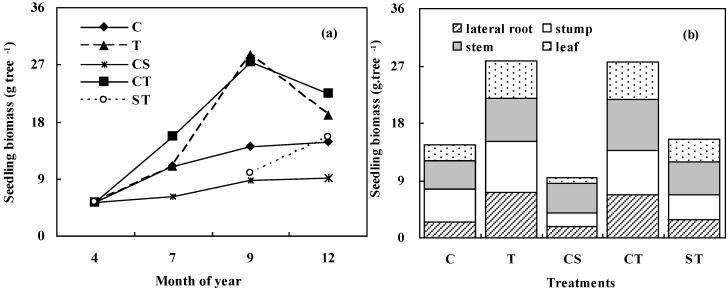
Changes in total biomass during the experimental period from April to December (**a**), and the biomass of each organ in December (**b**), for *K*. *paniculata* seedlings grown under the various treatments.

The total biomass of the *K*. *paniculata* seedlings, under all treatments, increased from planting time (April) until July, reached a maximum in September, and then remained relatively stable until December as shown in Figure 4a. The total biomass measured in December was highest for the seedlings grown in the CT treatment. The lowest total biomass was obtained under the CS treatment. Seedling biomass under was significantly different (*p* < 0.05) among the different treatments during the experiment period from July to December.

**Figure 5:**
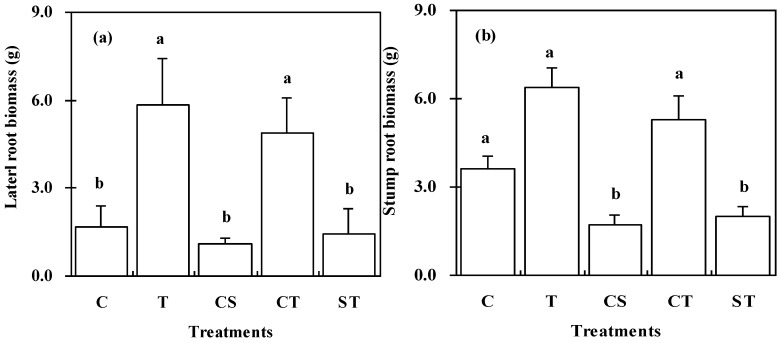

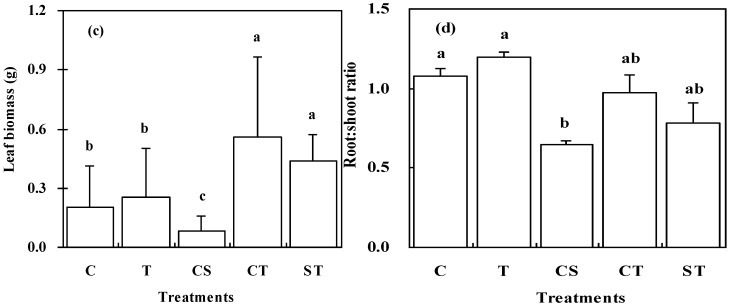
Harvest biomass comparisons for fine roots (**a**); root stumps (**b**); leaves (**c**); and root: shoot ratios (**d**) (means with error bars) of *K*. *paniculata* seedlings grown under the different treatments. Different letters indicate significant differences among the treatments (*p* < 0.05).

Leaf biomass shown in Figure 5cexhibited the same variation amongst the treatments as total biomass shown in Figure 4b, indicating that a positive relationship exists between leaf biomass and total biomass in *K*. *paniculata* seedlings. The values for lateral fine root biomass and for root stump biomass also differed significantly amongst the treatments. The highest values for both parameters were found in the seedlings grown in the T treatment. The lowest values were observed under the CS treatment.

The root -to- shoot ratios varied from 0.64 to 1.20 amongst the different treatments shown in Figure 5d. Higher values were obtained for seedlings grown in the T treatment with a lower value for seedlings under the CS treatment.

### 3.4 Heavy Metal Accumulation and Translocation

The concentrations of heavy metals measured in the shoots of the *K*. *paniculata* seedlings prior to the experiment averaged 20.87 ± 2.62 mg∙kg^−1^ for Mn, 417.08 ± 80.41 mg∙kg^−1^ for Fe and 62.70 ± 3.12 mg∙kg^−1^ for Zn, respectively. For the roots, the corresponding values were 31.59 ± 6.15 mg∙kg^−1^, 473.63 ± 83.02 mg∙kg^−1^ and 57.59 ± 3.41 mg∙kg^−1^, respectively.

**Figure 6:**
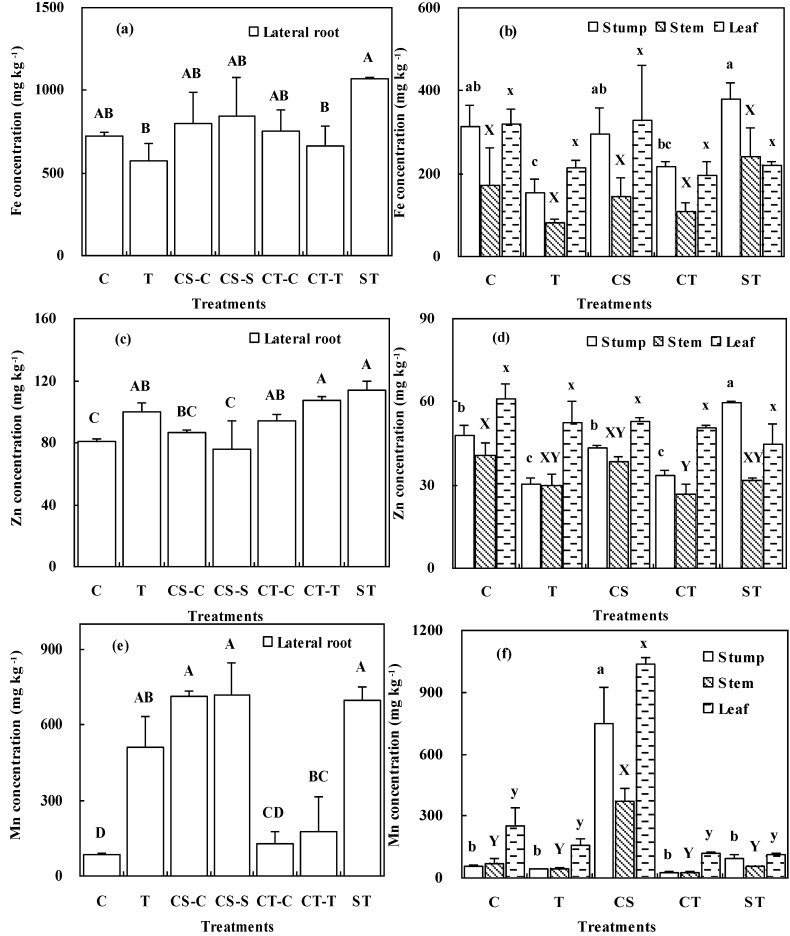
Concentrations of Fe (**a**,**b**); Zn (**c**,**d**); and Mn (**e**,**f**) in the different organs of *K*. *paniculata* seedlings grown under the various treatments. Mean values with error bars are shown. Different letters for a specific organ indicate significant differences among different treatments (*p* < 0.05). Capital letters (A, B, C and D) for lateral root, lowercase letters (**a**–**c**) for stump, capital letters (X and Y) for stem, and lowercase letters (x, y and z) for leaf.

In December, at the end of the experiments, the concentrations of heavy metals in the lateral fine roots of *K*. *paniculata* seedlings were found to vary according to both the heavy metal and the experimental treatment shown in Figure 6. Generally, the highest concentrations of Fe and Zn in the fine roots were found in those seedlings that had been growing in the ST treatment. In the case of Fe, the lowest concentration found in the fine roots was observed for the seedlings grown in mine tailings, and in the case of Zn the lowest concentration was found in the fine roots of soil-grown seedlings. In the case of the other treatments, the Fe and Zn concentrations found in the fine roots were intermediate and no significant differences were observed between the various treatments (Figure 6a,c). Growth in the CS treatment produced the highest Mn concentrations in the fine roots of the seedlings, while with the lowest value produced in the control treatment (Figure 6e).

For the root stumps, the highest Fe and Zn concentrations were found in seedlings grown in the ST treatment, and the highest Mn concentrations were found in seedlings grown under the CS treatment. The lowest Fe and Zn concentrations were found in the root stumps of seedlings grown in the T treatment. Root stumps from the other treatments showed no significant differences in the concentrations of the three heavy metals (Figure 6b,d,f).

Seedlings grown in all treatment showed no significant differences for Fe and Zn concentrations in the leaves (Figure 6b,d), but the values for Mn in leaves showed a different pattern from the values for Fe and Zn, and the highest concentrations were observed in the CS treatment (Figure 6f). No significant differences in leaf Mn concentration were found amongst the other treatments (Figure 6f).Of all the plant organs, the lowest heavy metal concentrations were found in the stems. With the exception of a significantly lower Zn content in the case of the CT treatment and a high Mn content for the CS treatment, no significant differences were observed amongst the various other treatments regarding individual heavy metal concentrations in the stems. Whole *K*. *paniculata* seedlings accumulated 2.24–3.94 mg of Fe, 0.41–0.97 mg of Zn and 0.96–6.00 mg of Mn. More than half of these accumulations of heavy metals were retained in the “below-ground” compartment shown in Table 2.

**Table 2:** Total accumulations of Fe, Zn, and Mn in *K. paniculata* seedlings growing under different treatments. The codes for the treatments abbreviated as C, T, ST, CT, and CS, each of them were described in detail in Section 2.2 *Experimental design and growth media*.

Treatments	Fe (mg)			Zn (mg)			Mn (mg)		
	Above-Ground	Below-Ground	Total	Above-Ground	Below-Ground	Total	Above-Ground	Below-Ground	Total
C	0.595	3.345	3.94	0.238	0.484	0.722	0.447	0.508	0.955
T	0.821	2.595	3.416	0.318	0.608	0.926	0.599	2.527	3.126
ST	1.58	3.695	5.275	0.283	0.523	0.806	0.668	1.588	2.256
CT	1.044	4.009	5.053	0.31	0.661	0.971	0.484	0.89	1.374
CS	0.563	1.68	2.243	0.198	0.211	0.409	2.59	3.414	6.004

## 4. Discussion

### 4.1. Differences in Characteristics of Mine Wastes and Soil

The two mine wastes displayed contrasting characteristics, which were consistent with the findings of other studies [29,39]. The high bulk density and nutrient concentration of mine sludge could be due to the Mn electrorefining process [38].

As shown in Table 1, compared to the control soils, Zn concentration in the mine tailings did not exceed the background values for local soils (85–117 mg∙kg^−1^; China National Environmental Monitoring Center, [41]) whereas the mine sludge contained higher Zn concentration than the background values. Nevertheless, none of the samples in this study contained a Zn concentration that exceeded the threshold value (200 mg∙kg^−1^) of Grade II (GB 15618-1995), the maximum tolerable value (300 mg∙kg^−1^) quoted by Sofianska and Michailidis [42], or the Zn concentrations in polluted soils (150–300 mg∙kg^−1^) reported by de Vries *et al.* [43].

Both mine tailings and mine sludge contained very high Mn concentrations, which were higher than the background concentrations found in the local soils [41]. The Mn concentrations presented in this study were also much higher than both the concentrations (2772 mg∙kg^−1^) found in soils from the Mn mine in Guangxi [31] and the maximum values (3637 mg∙kg^−1^) measured in a Mn mine in Morocco [44].

The pH values varied with the different soil substrates in this study. It was reported that an increase in soil pH value could reduce the plant-available concentrations of Zn and Mn in the soil [45] and affect the architecture of the whole root system [46]. Higher heavy metal concentrations in soils are related to more acidic (pH 5.0) soils [31], which affects heavy metal fractionation and its phytotoxicity such as Copper [47]. In this study, lateral roots developed better in the treatments T and CT than in the CS and ST treatments (Figure 5a, indicating the effects of the higher soil pH value. Consequently, the total biomass of seedlings in the T and CT treatments was greater than that in the other treatments.

### 4.2. Influences on Survival Rate and Fine Root Development

This result indicated that the presence of mine sludge reduced the survival rate–and reduced it to zero in the case of sludge alone. This phenomenon might be attributable to the unfavorable physical structure and the anoxic nature of Mn mine sludge [38], which therefore reduced the survival rate of the seedlings in the initial period of the experiment [37]. Nevertheless, the 50% survival rate observed for seedlings treated with the mixture of sludge and tailings is comparable with the survival rates that have been reported by Seo *et al.* [32] for tree species growing in mine wastes, supporting the conclusion that the manipulation of local mine wastes at our study site could increase the survival rate of tree seedlings.

The number of fine roots and their length are important indices of the degree of success of plant establishment on mine wastelands [26] because of their roles in the uptake of water and in the acquisition of nutrients from soil [48]. In this study, the treatments of T, S and ST were with higher Mn concentrations than that of the control soil. While the length of fine root was significantly greater in the T treatment than the treatments CT, CS and ST (Figure 3), which was related to the different soil pH values that correspondingly affect bioavailability of heavy metals in soils. From the perspective of soil nutrients such as N, P, and K, soil nutrients in the T treatment was not in the higher level in comparison with the treatments of C, S and ST (Table 1), which should not be the reason for the greater number of fine root and length of fine root in seedlings under the T treatment. Recently, López-Bucio *et al.* [49] reported that chromate activates the expression of low phosphate inducible reporter genes AtPT1 and AtPT2 in *Arabidopsis thaliana* transgenic seedlings and primary root growth was inhibited by 60% in seedlings upon exposure to 140 μM Cr(VI). And further studies confirmed that low Cr levels in *Arabidopsis* are beneficial to primary root length and lateral root growth, while toxic Cr concentrations activate a low-Fe rescue system which inhibits toxicity of Cr contaminated soils [50]. So the low bioavailability of heavy metal Mn concentration in the T treatment promoted the seedlings fine root growth due to its high soil pH value of 9.07.

### 4.3. Difference in Biomass Allocation of K. Paniculata Seedlings

The biomass and its allocation among tree organs have been used to examine the restoration effects of various tree species in different mine wastes, such as *Paulownia tomentosa* reported by Doument *et al.* [51], *Pinus densiflora*, *Robinia pseudoacacia* and *Amarpha fruticosa* by Seo *et al.* [32], and *Jatropha curcas* by Kumar *et al.* [37]. In this study, biomass and its allocation for *K. paniculata* seedlings varied with the different treatments. Higher biomass in lateral roots and in stump root (Figure 5a,b) resulted in higher total biomass in individual seedling (Figure 4b) as recorded in the treatments T and CT. These results indicate that developed fine root system should be the main factor of plant survival and high biomass. The mine sludge reduces the growth of *K. paniculata* seedlings on account of the high Mn concentration and its poor soil texture [38]. The total biomass values for *K. paniculata* seedlings grown in the ST treatment were close to those for seedlings grown in the control soil, indicating that mixing mine sludge with mine tailings in order to increase tree growth should be a feasible, low-cost option to promote phytoremediation in Mn mine wastelands.

Under the CT treatment and the CS treatment the lateral fine roots grew separately within the two halves of the plant pot, therefore it was possible in each of these cases to make a direct comparison of the lateral fine root biomass under the two conditions. This comparison indicated that soil provided the most favorable conditions for fine root growth, followed by mine tailings, whereas mine sludge presented the most unfavorable conditions for fine root growth.

Our values, known as root to shoot ratio, are higher than the field observations reported previously by Tian *et al.* [39] for planted trees (0.3), growing in the mine wastes of the Xiangtan Mn mine; and they are partly within the values (0.281 to 0.684) that have been recorded in subtropical forests, but they fall within the overall range of values recorded for shrubs (0.335 to 4.250) by Mokany *et al.* [47]. The general trend for root to shoot ratio to increase in line with increasing proportions of sand relative to clay, observed by Mokany *et al.* [52] in forests and woodlands, supports the findings in this study that, for Mn mine sludge, high bulk density allied to a clay soil texture are the major factors that inhibit tree re-vegetation.

### 4.4. Heavy Metal Accumulation and Translocation

Plants grown in heavy metal contaminated soils exhibit a variety of different heavy metal uptake or distribution mechanisms in order to be able to tolerate them [53]. In this study, with the exception of Mn in the CS treatment, lower Fe, Zn and Mn concentrations in the leaves than in the lateral roots suggests that *K*. *paniculata* adopts an exclusion strategy that confines heavy metals to the roots in order to avoid harming the leaves and damaging tree growth [53,54].

BCF and TF values are important in determining whether *K*. *paniculata* is a phytoextraction or a phytostabilization tree species [31]. The BCF for Fe ranged from 0.02 to 0.12 (Table 3). Most of the BCF values for Zn were higher than 1, which may be due to the Zn concerntration within the threshold value.

**Table 3:** Bio-concentration factor (BCF) and translocation factor (TF). Mean values are shown, with standard errors in parentheses, for Fe, Zn, and Mn of *K. paniculata* seedlings grown under different treatments.

Treatments		BCF (Mean ± SE)			TF (Mean ± SE)		
		Fe	Zn	Mn	Fe	Zn	Mn
C		0.03 (0.01)	1.39 (0.07)	3.12 (0.10)	0.44 (0.11)	0.76 (0.09)	2.54 (0.34)
T		0.12 (0.10)	1.18 (0.15)	0.01 (0.02)	0.37 (0.09)	0.53 (0.14)	0.19 (0.21)
CT	-C	0.02 (0.00)	1.11 (0.11)	0.25 (0.13)	0.26 (0.17)	0.54 (0.02)	0.71 (0.08)
	-T	−	2.90 (0.20)	0.01 (0.02)	−	0.47 (0.02)	−
S		−	−	−	−	−	−
CS	-C	0.03 (0.00)	1.04 (0.08)	1.38 (0.10)	0.41 (0.39)	0.61 (0.02)	1.41 (0.04)
	-S	−	0.32 (0.03)	0.06 (0.01)	−	0.70 (0.02)	−
ST		0.02 (0.01)	0.34 (0.05)	0.01 (0.01)	0.21 (0.05)	0.39 (0.10)	0.15 (0.10)

In the case of Mn, most of the BCF values shown in Table 3 were less than 1, which indicated that soil structure improvements achieved by mixing mine tailings and sludge with soil could increase BCF values.

With the exception of control soil, and the soil from the CS treatment, the other treatments all gave TF values of less than 1 (Table 3) for Mn, which proved that *K*. *paniculata* is the non-hyperaccumulator [23], implying that the heavy metals absorbed from the mine wastes were restricted distribution in the roots of the seedlings, and especially in the lateral roots. This kind of distribution is the most common resistance trait [26]. Plant species with BCF values larger than 1 are also considered to be hyperaccumulators [31,38]. Therefore, in terms of its BCF and TF values, *K*. *paniculata* is not a hyperaccumulator, but rather a Mn-tolerant tree species.

## 5. Conclusions

Our results show that *K*. *paniculata* has a high survival rate in mine tailings, but not in mine sludge, on account of the poor soil texture limitations of the latter. The enhanced seedling survival and rate of biomass growth seen in the mine sludge mixed with mine tailings indicates that local mine waste manipulation could improve soil structure and facilitate tree re-vegetation. The values of BCF and TF for the three heavy metals (Fe, Zn, and Mn) considered here were lower than 1, suggesting that *K*. *paniculata* is a Mn-resistant tree species suitable for phytostabilization in Mn mine wastelands.
